# Epidemiological analysis and comparison of 259 extensive burn patients from 2010 to 2021: Evidence for autonomous medical team

**DOI:** 10.1371/journal.pone.0346806

**Published:** 2026-04-21

**Authors:** Jingzhu Li, Yuanshu Wu, Yixin Wu, Xiaowan Fang, Zhenzhen Yan, Yuxiang Wang, He Fang, Xiaoyan Hu, Yicheng Ma, Shizhao Ji, Pengfei Luo, Chao Ji, Yongjun Zheng, Shichu Xiao

**Affiliations:** 1 Department of Burn Surgery, The First Affiliated Hospital of Naval Medical University, Shanghai, People’s Republic of China; 2 Department of Plastic Surgery, Xinhua Hospital, Shanghai Jiao Tong University School of Medicine, Shanghai, People’s Republic of China; Faculty of Medical Sciences of Minas Gerais, BRAZIL

## Abstract

**Background:**

This study aims to investigate the epidemiological and clinical characteristics of patients with extensive burns admitted to a burn center in eastern China from 2010 to 2021 and to evaluate the impact of an autonomous medical team implemented in 2016 on patient management.

**Methods:**

A retrospective study was conducted on patients admitted to the Burn and Trauma Center at the First Affiliated Hospital of Naval Medical University between January 1, 2010, and December 31, 2021. Data were compared and statistically analyzed using SPSS (version 26.0) for two periods: 2010–2015 and 2016–2021.

**Results:**

This study enrolled a total of 259 patients, with 106 cases spanning from 2010 to 2015 and 153 cases from the period of 2016–2021. Among them, there were a total of 198 male patients (76.4%) and 61 female patients (23.6%). The age range of the subjects varied between 1 and 90 years old, with a median age of 44 years (30 ~ 54 years). The distribution of burn total body surface area (%TBSA) ranged from 30% to 99%, with a median TBSA value of 50% (35% ~ 72%). Flame burns constituted the majority cause for extensive burns, accounting for 151 cases (58.8%), followed by scald burns comprising 51 cases (19.8%). Extensive burns predominantly occurred during the summer season. The limbs were identified as the most commonly affected areas, accounting for 146 cases (56.4%). The median length of hospital stay was recorded as 44 days (28-67.75 days), while the overall mortality rate stood at 7.34%. The proportion of middle-aged and elderly individuals increased in recent 6 years compared to earlier period. The incidence of flame and chemical burns decreased, while the severity exhibited an increase. There was a significant rise in the average number of surgical procedures performed during hospitalization. Meanwhile, the mortality rate experienced a decline without any notable variation in total length or cost associated with hospital stay.

**Conclusions:**

Our research findings suggest the need for dynamic adjustments in burn prevention measures based on changes in epidemiological characteristics, while also advocating for the establishment of autonomous medical teams with decision-making authority for critical care treatment of extensive burn patients.

## 1. Background

Burns continue to pose a significant threat to global human health and safety. According to statistics from the World Health Organization (WHO), burns claim the lives of 180,000 individuals annually, predominantly affecting low-income and middle-income countries. In China, burns are typically classified as extensive when they cover an area equal to or exceeding 30% of the total body surface area (TBSA), although regional variations in this definition may exist [1,2]. However, it is widely acknowledged that patients with extensive burns are highly susceptible to early post-injury shock, exhibit multiple complications, and experience a significantly elevated mortality rate [3,4]. Consequently, immediate intensive care intervention is imperative following the injury. Furthermore, due to the frequent necessity for repeated surgical procedures during later stages for wound repair, patients with extensive burns often necessitate prolonged hospitalization periods and incur substantial treatment expenses.

Existing literature reports that the global mortality rate for burn patients (including those with extensive burns) has been decreasing over the past few decades [5,6]. However, these reports primarily focus on epidemiological analysis of burn patients as a whole within specific regions, neglecting extensive burns population and lacking comparative examination of clinical data across different time periods to reflect advancements made by specialized physicians towards improving treatment success rates. Meanwhile, in the current burn treatment system in China, the management of extensive burn patients’ intensive care and wound care is often entrusted to a specialized medical team, even if these patients are initially admitted to an intensive care unit. In contrast, developed countries like Europe and America employ more advanced multidisciplinary medical practices where treatment teams comprise professionals such as burn surgeons, intensivists, nurses, nutritionists, physical therapists, and psychologists.

Therefore, this study aims to retrospectively analyze the epidemiological characteristics of extensive burn patients admitted to a level three burn center’s intensive care unit in Shanghai. This center serves as a leading facility for treating extensive deep burns in China with a total capacity of 17 beds dedicated to critically ill trauma patients including those with extensive burns. Annually, over 200 cases involving various critical illnesses are managed at this facility. In earlier years, patient care within the ICU was primarily overseen by individual attending physicians which often resulted in issues such as lack of coordination and standardized treatment plans. Since 2016, an independent attending physician group has been established at this center specifically responsible for managing ICU patients. By comparing data from two consecutive temporal periods within a specific time interval, this study aims to enhance public awareness regarding the dynamic epidemiological characteristics of burn injuries in the same region over time, and to provide indirect evidence for guiding the formulation and modification of targeted prevention strategies. The results also elucidate shifts in management concepts and strategies for patients with extensive burns at this center, thereby highlighting the significance of autonomous decision-making intensive care team in improving the success rate of treatment for patients with extensive burns.

## 2. Methods

### 2.1. Study design and oversight

Based on a comprehensive analysis of the overall patient situation spanning 12 years (2010 ~ 2021), patients were further categorized based on their year of admission: “first 6 years” for admissions between 2010 and 2015, and “last 6 years” for admissions between 2016 and 2021. The primary rationale for this subgrouping is the organizational shift in ICU management implemented since 2016, in which the intensive care medical team transitioned from an ancillary role—merely maintaining internal homeostasis and monitoring vital signs—to an autonomous authority with dominant decision-making power over the entire therapeutic strategy for critically ill patients. Since then, burn surgeons have been responsible only for operations and dressing changes, with any major surgical procedure requiring prior approval from the critical-care team. Treatment plans are jointly formulated by a multidisciplinary panel composed of dual-trained burn/intensivist physicians, specially trained ICU nurses, and specialists in nutrition, rehabilitation, endocrinology, and respiratory medicine. Perioperative care integrates individualized nutritional, metabolic, and analgesic protocols to achieve precise, one-stop management.

The age distribution, gender ratio, injury time, post-injury admission time, incidence of inhalation injury, as well as the gender ratio within each age group, causes of burns, burn area, treatment outcomes and length of hospital stay, total cost and other relevant information were meticulously examined and compared. The extraction of all clinical data was conducted by personnel specializing in data management within the information science department from 25/08/2025 to 30/08/2025, ensuring that personal identifiable information is anonymized to the maximum extent possible and sensitive data is strictly avoided. So, authors hadn’t access to information that could identify individual participants during or after data collection. Consequently, the necessity for informed consent is exempted. All individuals involved in data processing and analysis have received comprehensive ethics training to effectively minimize privacy risks. The present study was conducted in accordance with the guidelines outlined in the Helsinki Declaration of Human Medical Research Ethics and Good Clinical Practice, as established by the International Committee for Harmonization. This study was conducted with the approval of Shanghai Changhai Hospital Medical Ethics Committee (Approval No. CHEC2025−331).

### 2.2. Study population

The study enrolled extensive burn patients who were admitted to the Burn and Trauma Center of the First Affiliated Hospital of Naval Medical University for inpatient treatment between January 1, 2010, and December 31, 2021. These patients had a burn involving a total body surface area (%TBSA) equal to or greater than 30%. The first hospitalization occurs for all admitted patients following burns. Participants meeting any of the following criteria will be excluded from this study: 1) incomplete data entry in medical records; 2) hospitalization duration less than 24 hours; 3) initial hospitalization for scar or rehabilitation treatment subsequent to burn injury; or 4) multiple admissions of the same patient due to rehabilitation, scarring, or other reasons will only include the medical record from their first hospitalization after burn injury.

### 2.3. Data collection

The following data were collected from electronic medical records: (1) demographic information, including age, gender, admission date, and discharge date. Age was categorized into four groups: children (0 ~ 14 years), young adults (15 ~ 35 years), middle-aged adults (36 ~ 65 years), and elderly individuals (≥66 years). (2) injury occurrence pattern: The temporal distribution of injuries is as follows – spring (March to May), summer (June to August), autumn (September to November), and winter (December to February of the subsequent year). (3) clinical features include burn causes, burn area (%TBSA encompassing total burn area as well as second-degree and third-degree burns), burn location, presence of inhalation injury, and severity of burns. Burn causes are categorized into five groups: ‘flame burns,’ ‘scald burns,’ ‘electric shock injuries,’ ‘acid or alkali burns’ and ‘others.’ Burn area (%TBSA) is determined using the Chinese nine-point method with a 10% interval and classified into seven groups: 30% ~ 39.9%TBSA, 40% ~ 49.9%TBSA, 50% ~ 59.9%TBSA, 60% ~ 69.9%TBSA, 70% ~ 79.9%TBSA, 80% ~ 89.9%TBSA, 90% ~ 99.9%TBSA. Burn locations are divided into six categories: head/face/neck, upper limbs, lower limbs, trunk, hip, and perineum. If multiple locations are simultaneously burned, counting is performed for each location. Double-sided burns in the same location are counted once. Diagnosis of inhalation injury relies on fiberoptic bronchoscopy findings. The severity of burns is assessed using the revised Baux score which calculates age (in years) + total burn area (%TBSA) + (17 × inhalation injury), where age and total burn area represent actual values while inhalation injury is assigned a value of yes = 1 or no = 0. (4) treatment specifics encompassing the feasibility of endotracheal intubation or tracheostomy, surgical intervention rates, duration of hospitalization, and outcomes. Patient outcomes were classified as either death (including cases registered as automatic discharge due to death) or successful discharge (survival). The outcomes were based on records at the time of patient discharge.

### 2.4. Quality control

All personnel involved in this study have undergone comprehensive training in data processing and analysis techniques. The information has been extracted and preprocessed by the information science department, followed by a meticulous cross-checking and screening process based on medical record numbers conducted by two individuals for exclusion or inclusion purposes. Subsequently, the organized data undergoes further scrutiny from an additional pair of individuals who meticulously exclude any inadequate medical record data at a rate of 10%.

### 2.5. Statistical analysis

Data analysis was performed with SPSS 26.0 software. Descriptive analysis presented normally distributed continuous data as Mean ± SD, while non-normally distributed continuous data were represented as M (P25, P75). Count data were expressed as the number of cases (%). The gender ratio was indicated by a numerical ratio, with females assigned a value of 1. Between-group comparisons utilized independent samples t-test for normally distributed continuous variables and two independent samples Wilcoxon rank-sum test for non-normally distributed continuous variables. Categorical variables were analyzed using Chi-Squared test; Fisher’s exact probability method was employed when the sample size < 40 or expected frequency < 1; if Fisher’s exact probability method could not be calculated by SPSS for R × C contingency tables, Monte Carlo approximation method would be used instead. Pearson correlation coefficient was employed to describe linear relationships between two variables. Logistic regression analysis was performed to analyze nonlinear relationships between categorical variables and multiple independent variables. A significance level of *P* < 0.05 denoted statistical significance.

## 3. Results

### 3.1. Demographics

A total of 259 patients were included in this study, with 106 cases occurring within the first 6 years and 153 cases in the subsequent 6 years (Table 1). Among these patients, there were 198 males and 61 females, resulting in a male-to-female ratio of 3.237:1. The age distribution ranged from 1 to 90 years old, with a median age of 44 (30, 54) years. The majority of patients were young or middle-aged individuals (22.6% and 58.0%, respectively). There was no significant difference in gender ratio between the two six-year periods (χ^2^ = 3.230, *P* = 0.720, RR = 1.695, 95% confidence interval (CI) [0.950, 3.022]), but a significant difference was observed in age distribution (χ^2^ = 12.062, *P* = 0.007). Specifically, there was a decrease in the proportion of children and young adults (41.4% VS 29.4%), while an increase was observed among middle-aged and elderly individuals (55.8% and 2.9% VS 59.5% and 11.1%, Table 2).

**Table 1:** Epidemiological characteristics of 259 hospitalized patients with extensive burns (n = 259).

	Previous 6 years	Latter 6 years
	Median, IQR	
Age (years)	40(23.25, 47)	48(32, 57)
Burn area (% TBSA)	50(35, 75.25)	50(37.5, 70)
Total LOS (days)	45(26.5, 67.5)	42(25.5, 66)
ICU LOS (days)	27(9, 45)	20(7, 46)
Cost (yuan)	324187.28 (133956.7,651837.5350)	360201.95 (188007.36,731190.16)
Revised Baux score	99(78, 130.75)	110(88, 141)
	Number, Frequency (%)	
Gender (n, %)		
Male	75(70.8)	123(80.4)
Female	31(29.2)	30(19.6)
Age groups (n, %)		
Children	19(18.3)	11(7.2)
Youth	24(23.1)	34(22.2)
Middle-aged	58(55.8)	91(59.5)
Elder	3(2.9)	17(11.1)
Burn causes (n, %)		
Flame	71(67.0)	80(53.0)
Scald	20(18.9)	31(20.5)
Electricity	2(1.9)	4(2.6)
Chemical	5(4.7)	3(2.0)
Others	8(7.5)	33(21.9)
Season (n, %)		
Spring	27(25.5)	32(20.9)
Summer	43(40.6)	80(52.3)
Autumn	27(25.5)	31(20.3)
Winter	9(8.5)	10(6.5)
TBSA (n, %)		
30–39.9%	29(27.4)	40(26.1)
40–49.9%	21(19.8)	32(20.9)
50–59.9%	14(13.2)	20(13.1)
60–69.9%	6(5.7)	17(11.1)
70–79.9%	12(11.3)	12(7.8)
80–89.9%	8(7.5)	16(10.5)
90–99.9%	16(15.1)	16(10.5)
Burn location (n, %)		
Head/ Face/ neck	54(84.4)	77(86.5)
Upper limbs	60(93.4)	86(96.5)
Lower limbs	62(96.4)	84(94.5)
Trunk	60(93.4)	80(89.5)
Hip	38(59.4)	39(43.5)
Perineum	19(29.4)	20(22.5)
Inhalation injury (n, %)	52(49.1)	99(64.7)
Tracheal intubation (n, %)	7(6.6)	8(5.2)
Tracheostomy (n, %)	46(43.4)	70(45.8)
Outcome (n, %)		
Cured	93(87.7)	147(96.1)
Dead	13(12.3)	6(3.9)

Abbreviations: TBSA total body surface area, LOS length of stay, IQR interquartile range.

**Table 2:** Demographic characteristics of patients in two periods (n = 259).

Years	Patients	Gender (n, %)		Male/Female	Age group (n, %)			
		Male	Female		Children	Youth	Middle-aged	Elder
2010-2015	106^*^	75(70.8)	31(29.2)	2.419: 1	19(18.3)	24(23.1)	58(55.8)	3 (2.9)
2016-2021	153	123(80.4)	30(19.6)	4.100: 1	11(7.2)	34(22.2)	91(59.5)	17(11.1)
Total	259	198(76.4)	61(23.6)	3.237: 1	30(11.7)	58(22.6)	149(58.0)	20(7.8)

* In the first 6 years, 2 missing age group statistics data.

### 3.2. Epidemiological aspects and clinical characteristics

#### 3.2.1. Burn causes and the season of injury.

The case data provided in Table 1 indicates that among the patients admitted for large area burns over a span of 12 years, the predominant cause of injury is ‘flame burns’ (151 cases, 58.8%), followed by ‘scald burns’ (51 cases, 19.8%). These two factors also maintain their top positions in both the initial and latter six-year periods. A statistically significant difference exists in the distribution of injury causes between these two time intervals (*P* = 0.014): scald burns, electric shock injuries, and other factors have shown an increase in proportion during the later period, while flame burns and chemical burn cases have exhibited a decrease in proportion. Notably, scald burns account for a significantly higher percentage of injuries among pediatric patients (37.9%) compared to other age groups (Table 3).

**Table 3:** Distribution of injury factors in patients with extensive burns of different age groups over the 12 years (n = 259).

Age groups	Burn causes				
	Flame (n, %)	Scald (n, %)	Electricity (n, %)	Chemical (n, %)	Others (n, %)
Children	13(44.8)	11(37.9)	0(0.0)	1(3.4)	4(13.8)
Youth	39(68.4)	9(15.8)	3(5.3)	2(3.5)	4(7.0)
Middle-aged	88(59.1)	29(19.5)	3(2.0)	4(2.7)	25(16.8)
Elder	9(45.0)	2(10.0)	0(0.0)	1(5.0)	8(40.0)
Total	151(58.8)	51(19.8)	6(2.3)	8(3.1)	41(16.0)

In the analysis of the injured season, the highest proportion of patients with large-area burns treated in summer was observed (123 cases, 47.5%). However, no significant difference was found between the two periods at this point (χ^2^ = 3.468, *P* = 0.332). The distribution characteristics of age subgroups revealed that young children had the highest representation in spring (13 cases, 43.3%) and summer (9 cases, 30.0%). Young adults were most prevalent in summer (22 cases, 37.9%) and spring (19 cases, 32.8%). Middle-aged individuals accounted for a larger proportion during summer (81 cases, 54.4%) and autumn (35 cases, 23.5%), while elderly people were predominantly affected during summer (11 cases, 55.0%) and autumn (6 cases, 30%).

#### 3.2.2. Area, depth and severity of burns.

**Fig 1:**
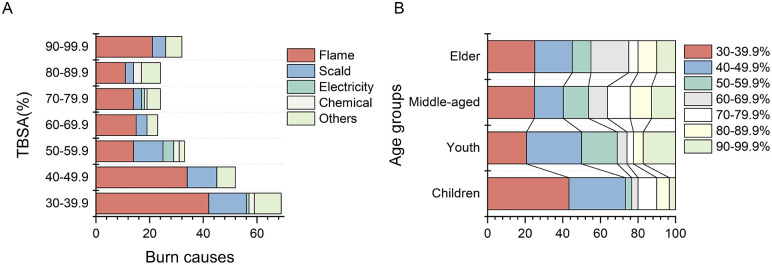
Distribution of burn causes and age among patients with extensive burns in different TBSA group.

The total body surface area (TBSA) distribution of the 259 patients ranged from 30% to 99%, with a median burn area of 50% (35%, 72%) TBSA. The majority of cases fell within the range of 30% to 39.9% TBSA (69 cases, 26.6%), followed by the range of 40% to 49.9% TBSA (53 cases, 20.5%). Other groups had between 20 and 40 cases each (Fig 1).

In terms of burn causes, patients hospitalized due to flame burns primarily exhibited a TBSA distribution between 30% and 50% (Fig 1). Scald burns and electric shock injuries resulted in burn areas mainly ranging from 50% to 60%. Burn areas caused by chemical burns were generally systemic, with a predominant range of 80% to 90%. The age distribution among patients reveals that burn areas for children and adolescents predominantly ranged from 30% to 50%, whereas other age groups exhibited burn areas exceeding 50%. There was no significant difference in burn area distribution across different age groups during the six-year period previous and latter (*P* = 0.077). Overall, second-degree burns covered an area ranging from 0 to 92% TBSA, with a median value of 30% (18%, 42%) TBSA. The distribution of third-degree burns was wider among included cases, covering an area ranging from 0 to 98% TBSA, and having a median value of 14% (4%, 40%) TBSA.

The median score of the revised Baux score in the included cases was 106 (83, 134), with a median score of 107 (87, 134) for male patients and 104 (72, 137.75) for female patients (Table 1). There was no statistically significant difference observed between the two groups (*Z* = −1.022, *P* = 0.307). The median revised Baux score for all patients was significantly higher in the later period, compared to the earlier period, with a Hodges-Lehmann median difference of 10.0 points (95% CI: 1.0 to 19.0, P = 0.032).

#### 3.2.3. Positions of burns.

The study encompassed a total of 679 burn sites, with the limbs emerging as the most frequently affected anatomical regions (both upper and lower limbs accounted for 146 each, constituting 56.4% of the cases). Other burn sites in descending order included the trunk (140 sites, 54.1%), head, face and neck (131 sites, 50.6%), hip (77 sites, 29.7%), and perineum (39 sites, 15.1%) (Table 1).

#### 3.2.4. Airway injury and intervention.

Among the 259 patients, inhalation injury was observed in 151 cases, resulting in an incidence rate of 58.3%. Notably, flame burns were identified as the primary cause of inhalation injury (54 cases, 36%). Tracheotomy was performed in a total of 101 cases (66.88%), while endotracheal intubation was conducted in 15 cases (9.93%) and both procedures were sequentially carried out in another 10 cases (6.62%). Furthermore, there were also 15 patients who underwent preventive or therapeutic tracheotomy due to deep neck burns or severe lung infections (Table 1).

#### 3.2.5. Number of surgeries.

The number of surgeries ranged from 0 to 34 for the 259 patients, with a median of 4 (1, 8). The median number of surgeries was significantly higher in the later six-year period than in the earlier period (4.0 (2, 9) vs. 3.5 (1, 6); Mann-Whitney U = 9488.5, P = 0.019). The Hodges-Lehmann estimate of the median difference was 1.00 operation (95% CI [0.00, 2.00]). Furthermore, there was a positive linear correlation between burn area and the number of surgeries performed (*r* = 0.428, 95% CI [0.316, 0.529], *P* < 0.001) (Table 1).

#### 3.2.6. Outcomes.

**Fig 2:**
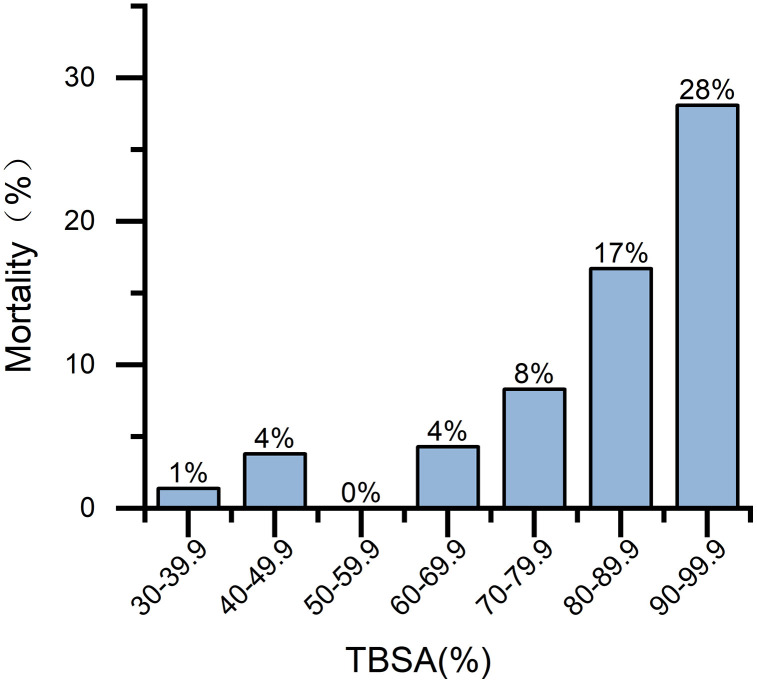
Mortality rate of hospitalized patients with extensive burns in different TBSA groups.

Among the 259 patients, 240 (92.66%) survived while 19 (7.34%) succumbed to their injuries. The age range of surviving patients spanned from 1 to 90 years old, with a median age of 44.00 (30, 54) years old. Male patients constituted the majority of fatalities (16 cases, 84.2%), ranging in age from 2 to 63 years old and having an average age of 40.32 ± 4.051 years old; middle-aged patients accounted for the highest proportion of deaths at a rate of 63.2% (12 cases), and no elderly patients died within this study group. Table 4 demonstrates that among deceased patients, the median total burn area was recorded as being at 85% TBSA (70%, 95%), predominantly distributed in >80%TBSA which accounted for 68.4%. There existed a statistically significant difference in outcome distribution across different TBSA groups (χ² = 23.822, *P* < 0.001). The mortality rate exhibited an upward trend as %TBSA increased (Fig 2). 16 out of 19 deceased patients were diagnosed with inhalation injury. The revised Baux score had a statistically significant impact on hospital outcomes (*P* = 0.027).

**Table 4:** Clinical characteristics of patients who died from extensive burns over the past 12 years (n = 19).

	First 6 years	Last 6 years
	Mean±SD	
Age (years)	39.85 ± 16.89	41.33 ± 20.88
	Median, IQR	
Burn area (% TBSA)	90(66.5, 96.5)	83(62.5, 95.75)
Revised Baux score	141(118.5, 155.5)	154.5(81.25, 165.5)
	Number, Frequency (%)	
Gender (n, %)		
Male	11(84.1)	5(83.3)
Female	2(15.9)	1(16.7)
Age groups (n, %)		
Children	1(7.7)	1(16.7)
Youth	4(30.8)	1(16.7)
Middle-aged	8(61.5)	4(66.6)
Elder	0	0
Burn causes (n, %)		
Flame	10	4
Scald	2	1
Electricity	0	0
Chemical	0	0
Others	1	1
Season (n, %)		
Spring	3	11
Summer	5	4
Autumn	4	1
Winter	1	0
TBSA (n, %)		
30–39.9%	1	0
40–49.9%	1	1
50–59.9%	0	0
60–69.9%	1	0
70–79.9%	1	1
80–89.9%	2	2
90–99.9%	7	2
Inhalation injury (n, %)	11(84.1)	5(83.3)
Tracheal intubation (n, %)	1(7.7)	2(33.3)
Tracheostomy (n, %)	13(100)	6(100)

*Abbreviations: TBSA* total body surface area, *LOS* length of stay, *IQR* interquartile range, *SD* standard deviation.

When comparing the clinical characteristics of deceased patients between the initial and subsequent 6-year periods, it was observed that there were 13 cases (12.3%) in the first 6 years and 6 cases (3.9%) in the latter 6 years. The disparity in outcomes between these two time frames exhibited statistical significance (χ^2^ = 6.411, *P* = 0.011, RR = 0.32, 95% CI [0.13, 0.81]). The attributes of deceased patients during different temporal intervals remained consistent with those of the overall patient population.

#### 3.2.7. Length of stay.

The duration of hospitalization for surviving patients varied from 1 to 476 days, with a median length of stay at 44 (28, 67.75) days. Among various age groups, the median lengths of hospital stays were as follows: children and adolescents 33.5 (12.5, 60) days; young adults 53 (36, 87）days; middle-aged adults 45 (31, 70) days; elderly individuals 30 (10, 47) days. There was a positive correlation between the extent of burn injury measured by %TBSA and the overall duration of hospitalization among surviving patients, especially in cases involving third-degree %TBSA burns. The median length of hospital stays among surviving patients was recorded at 45 (26.5, 67.5）day within the initial six-year period but declined slightly to 42 (25.5, 66) day over subsequent six years without any statistically significant difference observed between these time frames.

The length of ICU stay for surviving patients ranged from 1 to 145 days, with a median of 22 (7,46) days. The median length of ICU stay varied across different age groups: children and adolescents 15 (9, 33.5) days; young adults 28 (6, 47) days; middle-aged adults 29 (7, 48) days; elderly individuals 13.5 (7, 24) days. The relationship between length of ICU stay and %TBSA burn area in surviving patients aligns with the overall hospitalization duration. There were no significant differences observed in the length of ICU stay for surviving patients between the initial and latter six-year periods (Table 1).

#### 3.2.8. Costs.

The range of total hospitalization costs for the cases included in this study varied from 13,296.75 to 3,159,008.59 yuan, with a median value of 344,066.48 (170,276.72, 682,764.07) yuan. There was no statistically significant difference observed in the overall comparison of patients’ total hospitalization costs between the first and last six years (*Z* = −1.429, *P* = 0.153). However, there was a significant increase in total hospitalization costs during the latter six years compared to the preceding period specifically within the burn area group ranging from 30% to 39.9% TBSA (*Z* = −2.772, *P* = 0.006) (Table 1).

## 4. Discussion

An increasing number of studies have demonstrated that the incidence and mortality rate of burns worldwide have been consistently declining year by year, attributed to economic development, advancements in education and healthcare [2,5,7]. This positive trend is particularly pronounced in developed countries [2,7,8], while many developing nations still face significant medical, economic, and social burdens associated with burn injuries [9–11]. The objective of this study is to provide a comprehensive overview of the epidemiological characteristics and trends among extensive burn patients admitted to a level III burn trauma center’s intensive care unit in Shanghai from 2010 to 2021. In addition, to explore the potential role of a structural change in clinical management, we divided the 12-year period into two phases—before and after 2016—when an autonomous multidisciplinary intensive care team was formally established in our center, allowing for a comparative analysis of patient outcomes and care processes across these distinct organizational eras.

The male-to-female ratio of the included cases in this study was 3.237:1, which is significantly higher than the national gender ratio of 1.049:1 (National Bureau of Statistics, end of 2021) and slightly higher than most related research results [5,12–15]. These studies also found significant differences in burn incidence rates between genders, mainly due to males being more exposed to injury-causing factors resulting from social labor division, leading to a higher occurrence rate of extensive burns, consistent with our research findings. Although there was an increase in the male-to-female ratio comparing the first and last six years, it did not reach statistical significance. In recent years, there has been a decrease in children and young adults among patients while an increase in middle-aged and elderly individuals occurred. This shift may be attributed to society’s vigorous promotion activities for burn prevention targeting adolescents since especially after the Kunshan “8**·**2” explosion accident in 2014 when both government and society intensified their efforts on burn prevention education. This observation is supported by evidence from targeted prevention programs; for example, in northeast India, a comprehensive burn prevention program for students led to a significant reduction in both the incidence and severity of burns, along with improved awareness and retention of preventive knowledge among participants [16]. The rise in elderly burn cases may be linked to shifts in societal structure. As more women of reproductive age engage in the workforce, the traditional family roles related to childcare and household responsibilities have partially shifted to the elderly [17,18]. Coupled with the natural decline in self-protective capacity associated with aging, these factors collectively heighten the daily exposure risks faced by the elderly population.

During the summer, due to reduced clothing and increased exposure of skin, combined with the influence of environmental temperature on fire risk, it becomes a season characterized by a high incidence of large-scale burn cases in this study. This conclusion also consistent with the clinical recognition of most burn specialists. The current epidemiological investigation on burns lacks a consistent conclusion regarding the primary causative facto, yet thermal agents—specifically ‘flame’ and ‘hot liquid’—are consistently identified among the most common [5,8,15,19–21]. The distribution of burn causes by age group primarily indicates that children and adolescents predominantly experience ‘hot liquid scalds,’ while adults predominantly suffer from ‘flame burns.’ Possible contributing factors include: (1) Children and adolescents engage primarily in activities at home where they lack self-protection ability and sufficient awareness of danger. Accidental scalding from spilling hot liquids or improper heating measures (such as heating pads) is the main cause of injury [19,22–25]; (2) Adults have more opportunities for exposure to open flames or flammable materials due to work, labor, and family life. Therefore, adults have a higher proportion of injuries caused by ‘flame burns,’ including gas explosions during production or daily life [14,26–29]. In this study, ‘chemical burns’ accounted for a small percentage (3.1%), with the majority occurring on 80% ~ 90% TBSA (total body surface area), which is higher than previous literature reports [30–33]. We speculate that this may be mainly because our center is positioned as a higher-level specialized treatment center in East China region; therefore, patients transferred to our center after injury tend to exhibit more severe injury characteristics. However, there was a lack of relevant data on medical transportation collected in this study, and further research is needed to confirm this speculation.

Despite variations in daily activity environments among patients of different genders and age groups, they all exhibit a common characteristic regarding burn locations, primarily affecting the limbs. This phenomenon can be attributed to both the larger surface area of the limbs on the human body (approximately 63%) and their frequent involvement in daily activities, rendering them more susceptible to hazardous factors. It is crucial to emphasize that deep burns on the limbs may lead to an increased risk of scar hypertrophy and contracture at joint sites, posing significant threats to patients’ future life and work abilities. Therefore, it is imperative to enhance protective measures against potential burn risks in daily production and life while paying particular attention to safeguarding the limbs.

During the period from 2010 to 2021, our ICU observed a declining trend in the overall mortality rate of burn patients, with a decrease from 12.3% during 2010 ~ 2015 to 3.9% during 2016 ~ 2021. It is well-established that burn mortality rate is significantly influenced by factors such as age, severity of burns (including burn area and inhalation injury), severe shock, and infection [20,26,27,34]. In this study, we found a statistically significant impact of revised Baux score on patient outcomes. The proportions of concurrent inhalation injury among deceased patients remained consistent at 84.1% and 83.3% in the six years previous and latter, respectively, aligning with previous research findings [3,14,29,35]. The influence of gender on prognosis for severely burned patients remains controversial; among the nineteen deceased patients in this study, sixteen were male. Overall male patients exhibited a significantly higher mortality rate compared to females (8.1% VS 4.9%), but there was no significant difference between genders regarding revised Baux score. Although literature suggests that factors such as hormones and immune differences may act as protective factors against death risk for female critically burned patients [34,36,37], specific mechanisms and protective efficacy remain unclear due to limited sample size in our analysis concerning gender, %TBSA (total body surface area affected by burns), age, etc.

An unexpected and counterintuitive survival pattern was observed among elderly patients. Despite the increased proportion of middle-aged and elderly individuals in the latter period, none of the 20 elderly patients (≥66 years) in our cohort died during hospitalization, which contrasts with the typically poorer outcomes reported for this age group [38,39]. This finding warrants careful interpretation. First, our outcome was defined as survival status at discharge, and post-discharge mortality was not captured. Second, the median hospitalization cost for elderly patients was 244,124.96 yuan (IQR: 78,194.30–449,205.82). While slightly lower than the overall cohort’s median, this remains a substantial economic burden for many older adults with potentially limited financial resources. We speculate that some elderly patients might have been discharged earlier or transferred to lower-cost facilities due to an inability to afford continued hospital care, thereby not appearing as in-hospital deaths. The influence of socioeconomic factors on survival in critically ill patients is well-documented. For instance, Muangman et al. highlighted that the level of social support significantly predicts survival in burn patients, with survivors more likely to have adequate support than non-survivors [40]. Furthermore, approximately 46% of elderly heart failure patients have delayed seeking medical care due to financial constraints [41]. In the United States, even insured adults often face severe financial hardship after emergency surgery [42]. Thus, economic pressures may have led some high-risk elderly patients to exit our healthcare system before in-hospital death could occur, creating the appearance of “zero mortality” in this subgroup.

**Fig 3:**
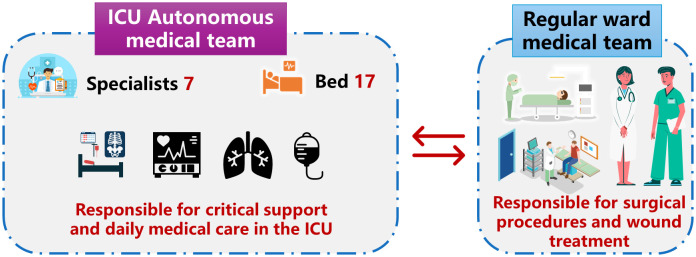
A novel management model for patients with extensive burns..

Team collaboration is a defining feature of modern burn care, with its model having evolved from a “multidisciplinary” to a “transdisciplinary” approach [43,44]. Early teams were typically led by burn surgeons, with other members providing parallel support within their respective professional domains, representing the sum of disciplinary contributions. In contrast, transdisciplinary teams achieve synergistic effects—where the whole is greater than the sum of its parts—through frequent interaction, integration of perspectives and skills, and the formation of a unified, collaborative entity [45]. This trend toward transdisciplinarity has demonstrated positive outcomes in pediatric burn care [43]. The practice within our study serves as a concrete example of an effective transdisciplinary team in action. The early monitoring and treatment of extensive burn patients in this center underwent a transition over the latter six years, with an independent intensive care medical team assuming responsibility for these tasks. During intensive care treatment, treatment decisions for extensive burn patients are made by an independent team of critical care specialists, including physicians selected from burn specialists and trained in critical care medicine (Fig 3). Nurses in the intensive care unit also undergo specialized training, and consultations are sought from professionals in nutrition, rehabilitation, endocrinology, gastroenterology, and respiratory medicine. Dedicated burn doctors assume responsibility for surgical procedures and daily wound dressing for patients. Early surgical decisions for critically ill burn patients must be approved by the critical care team. They collaborate to develop treatment plans based on individual patient conditions and provide comprehensive medical care to ensure optimal treatment outcomes and patient recovery. This multidisciplinary teamwork approach facilitates the provision of comprehensive and personalized treatment plans to address the complex medical needs of extensive burn patients. Compared to the statistical data of the previous period, patients admitted in the subsequent 6 years exhibited more severe burn injuries (higher revised Baux score) and underwent a greater number of surgeries. However, there was no significant increase observed in the total length of hospital stay or overall cost. Despite the recognized importance of multidisciplinary teams, their independent establishment remains challenging. Evidence suggests that external mentorship can critically support this process by providing structured guidance [46]. The framework presented in this study offers a transferable model to facilitate the development of functionally integrated burn care teams.

Due to economic development and government healthcare policies, this study found no significant difference in total hospitalization costs for burn patients between two time periods when inflation rates were not considered. Interestingly, there was a notable increase in total hospitalization costs for patients with burn areas of 30% ~ 39.9% TBSA during the latter six years compared to the previous period (*P* = 0.006). This increase may be attributed to the widespread utilization of biological dressings, functional dressings, and novel disinfectants (such as guanidine-based disinfectants) on burn wounds. In order to gain further insights into this matter, future research should focus on conducting a cost-effectiveness study regarding survival rates among patients with extensive burns.

Additionally, it should be noted that while short-term fluctuations in-patient admissions may occur due to uncertain factors such as major industrial accidents, the literature reports a consistent downward trend in overall burn incidence [47]. This can be attributed to the widespread dissemination of government knowledge on production, life and fire safety, as well as burn prevention measures. However, the future advancement of this specialized discipline, particularly in terms of conducting high-quality clinical research, faces numerous challenges. Therefore, when constructing new specialties within this field, it is crucial to consider potential adverse influencing factors.

This retrospective study was conducted at a single center in Shanghai, which may introduce potential limitations and biases to the results. Firstly, the limited sample size reduces statistical power and increases the risk of type II errors, particularly for subgroups with insufficient cases for effective multivariate statistical analysis. Even in subgroup analysis by gender, the number of female patients (61 cases) is inadequate to achieve sufficient statistical power due to the disproportionate effects of burn area, age, and inhalation injury on burn outcomes. Secondly, as most hospitalized patients in this center originate from Shanghai and surrounding provinces such as Jiangsu, Zhejiang, Anhui etc., generalizing these findings to other regions or institutions should be done cautiously. The rapid establishment and development of burn centers across various economically developed areas have led to both significant benefits in nationwide burn treatment but also introduced potential bias in patient admission at this center. Thirdly, this study only focuses on epidemiological characteristics of patients with extensive burns who were admitted after burns; thus, it cannot fully reflect the overall trend during this period due to deaths occurring at the scene where injuries were too severe. Fourth, Our study design cannot establish causality for the observed outcome improvements. The significant decrease in mortality coinciding with the implementation of the autonomous ICU team may also be influenced by 1) the non-contemporaneous comparison bias, as numerous other advancements in therapy, diagnostics, and general care occurred over the 12-year period; and 2) other unmeasured factors such as changes in referral patterns, which were not fully adjusted for in our analysis. Lastly, inherent limitations associated with retrospective studies result in missing clinical characteristic data from databases including information regarding injury location and direct cause of death.

## 5. Conclusions

This study reveals that the majority of patients admitted to this center with extensive burns are middle-aged and young adults, predominantly male. Flame burns are the primary cause of injury, with a higher incidence during the summer months, particularly affecting the limbs. To effectively prevent burn injuries in the future, it is crucial to focus on these demographic characteristics and prioritize education on protective measures for adult males in both their occupational and daily lives. This will enhance their awareness of burn risk factors and contribute to reducing the incidence rate within this population group. Achieving this objective necessitates collective participation and concerted efforts from society as a whole.

The severity of burns emerges as a clear prognostic risk factor for patients with extensive burns. Burn specialized centers play an indispensable role in treating such patients; therefore, we strongly recommend integrating current technological advancements and clinical treatment methods by adopting more individualized assessments of burn mortality risks along with early recognition of post-injury severe shock, inhalation injury, and systemic infections upon admission. These measures will further mitigate the risk of death among patients with extensive burns, significantly improving successful treatment rates.

Overall, our research reports a significant improvement in survival rates for extensive burn patients over a 12-year period, during which a more structured, autonomous, and multidisciplinary intensive care model was implemented. We suggest that specialized burn centers consider evaluating similarly integrated, team-based approaches to care coordination, while maintaining a focus on primary prevention targeted at high-risk populations.

## Supporting information

S1 File Databases. (XLSX)

## Abbreviations

SD: standard deviation
TBSA: total body surface burn area
